# The Productivity Costs of Premature Mortality Due to Cancer in Australia: Evidence from a Microsimulation Model

**DOI:** 10.1371/journal.pone.0167521

**Published:** 2016-12-12

**Authors:** Hannah E. Carter, Deborah J. Schofield, Rupendra Shrestha

**Affiliations:** Faculty of Pharmacy, University of Sydney, Sydney, Australia; Old Dominion University, UNITED STATES

## Abstract

**Aim:**

To estimate the productivity costs of premature mortality due to cancer in Australia, in aggregate and for the 26 most prevalent cancer sites.

**Methods:**

A human capital approach was adopted to estimate the long term impacts of Australian cancer deaths in 2003. Using population mortality data, the labour force participation and the present value of lifetime income (PVLI) forgone due to premature mortality was estimated based on individual characteristics at the time of death including age, sex and socioeconomic status. Outcomes were modelled to the year 2030 using economic data from a national microsimulation model. A discount rate of 3% was applied and costs were reported in 2016 Australian dollars.

**Results:**

Premature deaths from cancer in 2003 resulted in 88,000 working years lost and a cost of $4.2 billion in the PVLI forgone. Costs were close to three times higher in males than females due to the higher number of premature deaths in men, combined with higher levels of workforce participation and income. Lung, colorectal and brain cancers accounted for the highest proportion of costs, while testicular cancer was the most costly cancer site per death.

**Conclusions:**

The productivity costs of premature mortality due to cancer are significant. These results provide an economic measure of the cancer burden which may assist decision makers in allocating scare resources amongst competing priorities.

## Introduction

Cancer accounts for 8.2 million deaths and 196.3 million years of healthy life lost globally each year [1]. Despite improving the survival rates, the occurrence of cancer is expected to continue to increase due to population growth and ageing, as well as the increasing prevalence of established but preventable risk factors including smoking, overweight and physical inactivity [2].

While the costs imposed by cancer on the health care system are well documented, there is an increasing recognition of the impact of cancer on economic productivity. The productivity costs associated with premature mortality as a proportion of the total costs of cancer have been found to be significant [3, 4], and a number of recent studies have estimated the productivity costs of cancer mortality across countries in Europe, the US and Asia [3–11]. These studies have produced wide ranging estimates of productivity losses of between $28,569 per uterine cancer death in Puerto Rico [12] to $1.3 million per testicular cancer death in the US [5].

A 2014 review of studies calculating premature mortality losses from cancer in 2000–2013 revealed the lack of cancer sites assessed to be a major gap in the evidence base, with most studies focussing on a single or limited number of the most common cancer sites. There was also a predominance of studies from Europe and the US, with limited evidence from other countries. The review concluded that comprehensive, standardised estimates of premature mortality losses in different settings are needed if these measures are to be useful in assessing the societal cancer burden.

The aim of this paper is to address this gap in the evidence by estimating the productivity costs associated with premature deaths due to cancer in Australia. We use evidence from a microsimulation model to quantify the economic impacts across all cancers, as well as the 26 most prevalent cancer sites, using a consistent and rigorous methodology. Outcomes were modelled to the year 2030 and are reported in terms of the working years and present value of lifetime income (PVLI) lost due to mortality. Distributional analyses of these impacts by age and sex are also presented.

## Methods

We adopted the human capital approach to estimate the potential economic gains forgone due to premature cancer related mortality. This approach is based on the premise that, in the event a death of an individual could have been prevented, the individual would go on to live a typical life, earning income and contributing to the nations productivity [13]. This is the traditional method for valuing the productivity related costs of illness [14], and it remains the dominant approach in the recent literature [6].

The long term costs of cancer mortality were extracted from LifeLossMOD, a previously developed microsimulation model to estimate the economic impacts of all cause premature mortality. The process by which LifeLossMOD was developed is described in detail in Carter et al [15] and is summarised below. For the purpose of this study, cancer deaths were extracted from the model based on the WHO International Classification of Diseases, 10th revision (ICD-10) code recorded as the underlying cause of death on the official death certificate (codes C00–97) [16]. A full list of the ICD-10 codes associated with each cancer type reported is included in S1 Table.

### Developing the model

**Fig 1:**
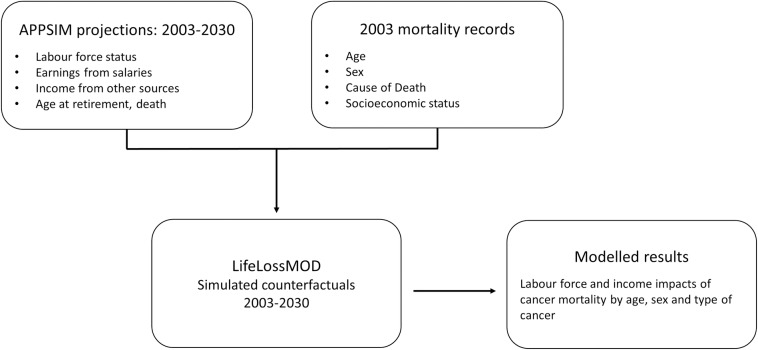
LifeLossMOD: Structure of the model and relationships between key data items

The structure of the model is outlined in Fig 1. LifeLossMOD estimates the potential economic gains forgone due to premature mortality by assigning a counterfactual lifespan to each individual that died prematurely in 2003. A premature death was defined as occurring before the age of 80 years, which was close to the Australian life expectancy [17]. The model was based around a population mortality dataset that contained information on the age, sex, cause of death and socio-economic status for every individual whose death was recorded in that year [18].

The process of assigning counterfactual lifespans to each cancer death was based on the premise that individuals with the same demographic characteristics could be assumed to experience similar life patterns and events including hours worked, income earned and retirement age over a period of time into the future. A counterfactual death could occur at any given age, and was modelled based on the age, sex and socioeconomic status of an individual at the time of their premature death in 2003. Information on these life patterns was obtained from a separate microsimulation model, APPSIM.

APPSIM is a dynamic model of the Australian population that was developed in collaboration with the Australian government with the aim of evaluating the impact of future fiscal and social policies [19]. The model uses a one percent sample of the 2001 Australian Census (188,000 records) as its base population. Large longitudinal survey datasets [20, 21] and official demographic data and projections [22] were used to generate transition probabilities for events within the model. These probabilities were then applied to the cohort of individuals within the model to annually update the population’s characteristics up to the year 2030.

LifeLossMOD was then able to match individuals in the 2003 mortality dataset at random with similar individuals in the 2003 APPSIM population based on each available combination of age category, sex and the socioeconomic status. To allow for the effects of uncertainty in the matching of records, the process was replicated 100 times to create 100 unique datasets. These 100 simulated datasets are what comprises LifeLossMOD. The results contained throughout this paper report the mean of the 100 datasets. Where present, 95% confidence intervals have been calculated using the percentile method.

### Estimating the productivity impacts of premature mortality in 2003

The projected labour force participation forgone due to premature cancer related mortality in 2003 was estimated by accumulating the weekly hours worked by each individual in the model over the period from 2003 to 2030. For each individual, a full time equivalent working year was derived by dividing the accumulated number of hours worked by the number of hours in a standard Australian working year [23].

The productivity costs of premature cancer related mortality were estimated by deriving the Present Value of Lifetime Income (PVLI) forgone. The PVLI represents the lifetime stream of private income an individual is expected to earn, valued in today’s currency (thereby excluding the effects of inflation). The estimate includes earnings from wages and salaries as well as private income generated from other sources including business profits and investments. Transfer payments were excluded to avoid double-counting on a macroeconomic level. The modelled income was estimated on an annual basis for each individual in the model, taking into account a range of factors including the individual’s age, sex, labour force participation and life expectancy. Total income was assumed to grow at a rate of 1% per annum above inflation [24] and a discount rate of 3% was applied. The resulting incomes were inflated from AUD 2010 to AUD 2016 using the national Consumer Price Index [25].

## Results

Table 1 provides a summary of the total number of premature cancer related deaths included in this study by age and sex, as well as the counterfactual years of life lost (YLL) between 2003 and 2030. Male deaths accounted for 58% of the total number of deaths and 56% of the total years of life lost. Deaths occurring between the ages of 65 and 80 accounted for the majority of deaths in both men and women.

**Table 1:** Number of deaths and YLL from premature cancer-related mortality in 2003.

Age	Male		Female		Total	
	Deaths in 2003	Counterfactual YLL to 2030	Deaths in 2003	Counterfactual YLL to 2030	Deaths in 2003	Counterfactual YLL to 2030
<15	54	1,261	35	804	89	2,064
15–24	57	1,345	42	980	99	2,326
25–34	121	2,791	153	3,568	274	6,359
35–44	422	9,783	524	12,396	946	22,179
45–54	1,338	31,069	1,376	32,928	2,714	63,997
55–64	3,385	73,828	2,497	56,708	5,882	130,536
65–80	9,674	141,598	6,055	100,115	15,729	241,713
**Total**	**15,051**	**261,675**	**10,682**	**207,499**	**25,733**	**469,174**

YLL = years of life lost

**Fig 2:**
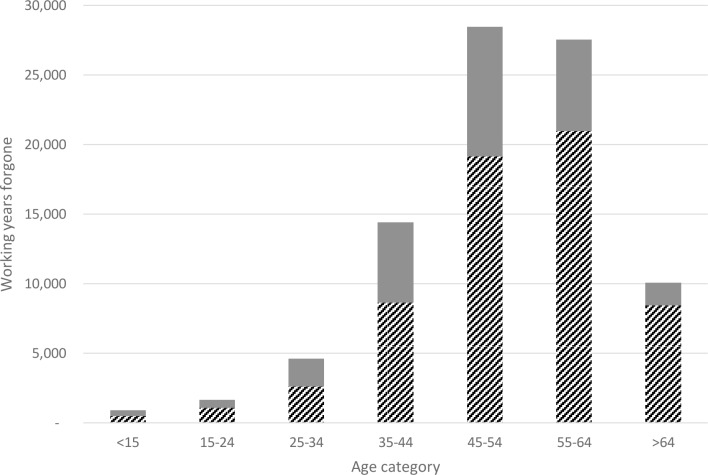
Working years forgone due to premature mortality from cancer in 2003, modelled to the year 2030. Hatched bar, males; solid bar, females.

The labour force analysis revealed that a total of 88,000 working years were lost due to premature cancer deaths occurring in 2003, with male deaths accounting for 61,000 working years and female deaths accounting for 27,000 (Fig 2). Deaths occurring between the ages of 45 to 64 years were responsible for over 60% of the number of working years lost for both men and women.

The PVLI lost due to cancer deaths in 2003 was estimated to be $4.20 billion (95% CI $4.14–$4.26 billion) over the period between 2003 and 2030 (Table 2). Male deaths accounted for 74% of the total PVLI lost, which was a function of the higher number of premature deaths among men (Table 1), their higher labour force participation (Fig 2), and their higher average incomes [26]. Deaths occurring between the ages of 35 and 64 accounted for 82% of the total PVLI impact.

**Table 2:** PVLI lost due to premature mortality in from cancer in 2003, by age and sex.

	PVLI ($ millions)	95% CI	% of total
**Male**			
<15	14	42,675	0%
15–24	45	40–51	1%
25–34	142	126–152	3%
35–44	505	505–487	12%
45–54	1,084	1,054–1,113	26%
55–64	950	923–975	23%
65–80	388	373–407	9%
Total	3,128	3,082–3,178	74%
**Female**			
<15	17	14–21	0%
15–24	22	18–26	1%
25–34	79	70–87	2%
35–44	233	217–246	6%
45–54	382	362–400	9%
55–64	269	256–287	6%
65–80	68	63–75	2%
Total	1,071	1,046–1,106	26%
**All**	4,200	4,140–4,258	**100%**

PVLI = Present value of lifetime income; CI = confidence interval

The PVLI lost due to premature mortality by cancer site was estimated (Table 3). Lung cancer, followed by colorectal cancer, were found to be responsible for the greatest loss in PVLI, together accounting for 30% of the total PVLI impact. Testicular cancer was found to be the most costly cancer by site with each death resulting in an average PVLI loss of $793,000. This was followed by bone and connective tissue cancers ($368,000 per death) and brain cancer ($325,000 per death).

**Table 3:** Cumulative GDP impacts of premature mortality (2003 to 2030).

Cancer type	Working years lost	PVLI lost ($ millions)	95% CI	% of total PVLI lost	No. of deaths	PVLI lost per death ($000’s)
Lung cancer	15,943	765	738–785	18%	5,746	133
Colorectal cancer	10,332	497	471–516	12%	3,253	153
Brain cancer	6,571	326	309–340	8%	1,003	325
Breast cancer	7,519	307	293–324	7%	2,171	142
Melanoma	4,897	249	239–265	6%	867	288
Lymphoma	4,263	209	193–221	5%	1,101	190
Leukaemia	4,180	201	190–213	5%	990	203
Pancreatic cancer	4,083	197	187–207	5%	1,398	141
Oesophageal cancer	3,134	156	145–167	4%	849	184
Stomach cancer	3,113	154	145–165	4%	830	186
Liver cancer (excluding hepatitis B and C related)	2,929	150	139–158	4%	720	209
Mouth and oropharynx cancers	2,805	142	134–151	3%	566	250
Prostate cancer	2,605	125	115–135	3%	1,547	81
Kidney cancer	2,414	121	114–130	3%	656	185
Bone and connective tissue cancer	1,890	88	79–99	2%	240	368
Ovarian cancer	1,505	61	55–68	1%	632	97
Multiple myeloma	1,205	58	52–65	1%	488	120
Bladder cancer	1,029	50	44–56	1%	480	104
Cervical cancer	967	39	33–45	1%	213	183
Laryngeal cancer	718	36	31–42	1%	186	192
Non-melanoma skin cancers	639	32	26–37	0.8%	197	163
Gallbladder cancer	544	26	22–30	1%	192	134
Corpus uteri cancer	354	14	43,770	0.3%	183	79
Testicular cancer	237	13	10–16	0.3%	16	793
Eye cancer	126	7	4–9	0.2%	24	275
Thyroid cancer	96	4	3–6	0.1%	54	78
Other malignant neoplasms	3,554	171	158–184	4%	1,131	151
All	87,653	4,200	4,140–4,258	100%	25,733	163

GDP = Gross Domestic Product; PVLI = Present Value of Lifetime Income; CI = confidence interval

**Fig 3:**
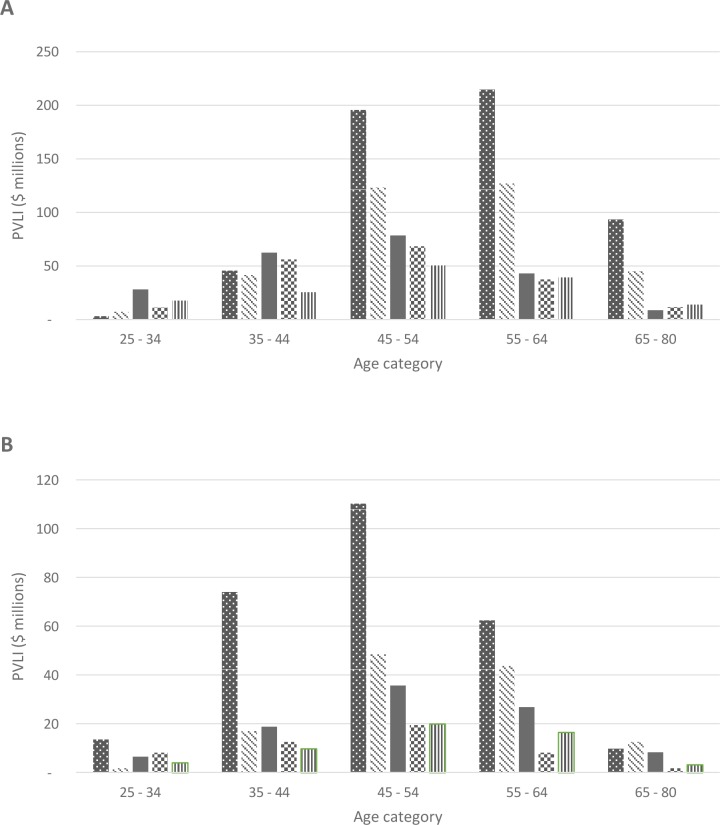
Cancer sites with the highest present value of lifetime income (PVLI) forgone, adults age 25–80 at the time of death in the year 2003. A) Men. From left to right: Lung cancer, colorectal cancer, brain cancer, melanoma, lymphoma. B) Women. From left to right: Breast cancer, lung cancer, colorectal cancer, brain cancer, ovarian cancer.

The diseases associated with the highest total PVLI loss for both men and women were assessed across ten year age categories (Fig 3). For men aged under 45, brain cancer resulted in the greatest loss in PVLI ($101 million). Lung cancer had the highest impact in men aged 45 to 80 ($504 million) followed by colorectal cancer ($295 million). Melanoma and lymphoma were the fourth and fifth most costly cancers overall. In women, the PVLI lost due to breast cancer dominated the cost of all other cancers for women aged 25 to 64 ($260 million). The next most costly cancer sites for women in terms of the total PVLI loss were lung, colorectal, ovary and brain.

Full individual level datasets summarising the modelled working years (S2 Table) and PVLI forgone (S3 Table) due to premature cancer mortality by age, sex and cancer type is contained in the Supporting Information files.

## Discussion

This is the first study to estimate the productivity costs of cancer mortality in Australia. Using a complete population mortality dataset, we applied a consistent methodology to generate estimates of productivity costs across all major cancer sites and many less prevalent sites. This enabled highly reliable comparisons of the relative productivity impacts across cancer sites as well as various combinations of age and gender. When projected to 2030, premature deaths from cancer in 2003 were responsible for a total loss of 88,000 full time working years and $4.2 billion in private income forgone.

Our results indicate that the productivity costs associated with cancer sites are broadly related to the number of deaths, with lung and colorectal cancers together accounting for 35% of deaths and 30% of the total PVLI impact. Notable exceptions include cancers where deaths typically occur in a younger and/or predominately male cohort, which can be explained by the higher productive capacity of these individuals into the future. Specifically, we estimated large PVLI losses per death for testicular ($793,000), bone and connective tissue ($368,000) and brain ($325,000) cancers relative to the average loss of $163,000 per premature cancer death. This compares to the Australian full time adult average annual ordinary time earnings of $77,168 in 2015 [26]. Conversely, while prostate cancer was the fourth most common cause of premature cancer death, it was ranked fourteenth in terms of the total PVLI loss. This can be explained by the lower proportion of deaths in men of working age, with 75% of all prostate cancer deaths occurring in men aged 75 and over [27].

The difference in the projected working years lost between men (61,000 years) and women (27,000 years) was notable, and is consistent with results reported elsewhere [6]. Some of this difference can be explained by the respective number of deaths, with approximately 50% more premature cancer deaths recorded in men as opposed to women in 2003 (Table 1). However, differences in labour force dynamics also contribute to this effect. In 2003, the labour force participation rate for Australian men was 71.3%, compared with 55.8% in women [28]. While the APPSIM projections we have applied account for the expected increase in female labour force participation into the future, they also account for projections that women will continue to be more likely to work part time [29].

Relative to other causes of death, the burden of cancer mortality is high. Cancer contributes more to the fatal burden of disease in Australia than any other disease category, accounting for 34% of the total years of life lost [30]. Results from LifeLossMOD indicate that cancer accounts for 30% of the PVLI loss associated with all-cause premature mortality [31].

A growing body of work estimating cancer-related productivity losses has developed in recent years, with a particular focus on premature mortality costs. In the US, Ekwueme et al estimated that the total premature mortality cost for all cancers in 2006 was $US 173.1 billion, equating to 216,701 USD per death. In Europe, these costs have been most recently estimated at €75 billion in 2008, equating to an average productivity cost of €219,241 per cancer death [7]. Significant variation in the cost per death between countries were reported, ranging from a high of €616,230 in Switzerland to a low of €51,925 in Bulgaria.

While most previous studies have produced estimates of productivity costs for all cancers or a single or small number of cancer sites, we identified two methodologically comparable studies detailing productivity costs across a large number of cancer sites. Bradley et al estimated cancer related productivity costs across the 19 most common cancer sites in the US [5], while Hanly et al estimated costs by site for all cancers across Europe [7]. The distribution of productivity impacts reported in these studies was largely consistent with our findings, with some exceptions. While lung cancer deaths were responsible for the highest total productivity cost across all studies, these costs made up a higher proportion of the total PVLI loss in the US (27%) and Europe (23%) relative to our estimate for Australia (18%). Deaths from colorectal and breast cancers were also associated with consistently high PVLI losses across studies, ranging from 8–12% of the total PVLI impact. Australia was the only country where melanoma was included in the top five most costly causes of death. Brain cancers in Australia were responsible for a relatively higher PVLI loss than those reported for Europe and the US. When estimating the cost per death, testicular cancer emerged as the most costly cancer site across all studies.

While these comparisons suggest a broad level of consistency in the distribution of productivity costs across cancer sites, it is evident that there are limitations in comparing studies across countries due to differences in the pattern of cancer deaths. This is further exacerbated by differences in the methodological approach across studies. As noted by Hanly et al in their recent review, key areas of difference include the method for valuing production losses, the inclusion of unpaid labour costs, and explicit adjustment for economic variables such as labour force participation rates, unemployment rates and wage growth rates [6]. The review concluded that there is a need to derive comprehensive estimates of productivity losses associated with different cancers, in different settings.

The strength of the microsimulation method we have applied was its capacity to project outcomes on an individual level. This is a key advance over previous studies, allowing for enhanced complexity and variability to be introduced into the analysis. For example, while previous studies have been limited to projecting the productivity costs of mortality based on assumptions around average wage rates, labour force participation and retirement ages, the estimates we report are able to account for the socioeconomic factors which influence an individual’s income and hours worked. In addition, the model incorporated projected trends in wage growth, labour force participation and actual retirement age, the latter of which is particularly important given the majority of premature cancer deaths occur beyond the traditional retirement age.

The microsimulation approach also allows for the assessment of a broader scope of information. Previous studies have calculated a Present Value of Lifetime Earnings (PVLE) estimate to determine the net productivity loss to society, which is based on salary-related earnings alone. We present a Present Value of Lost Income (PVLI) estimate which is able to account for all private income, including profits from business and other investments.

It is important to note that in addition to its mortality impact, cancer is also responsible for significant morbidity with associated productivity costs including the need for time away from work for both patients and their carers’. The analysis we have presented estimates the productivity related costs of cancer mortality alone, and as such cannot be considered representative of the full productivity burden of cancer.

The use of the human capital approach has its limitations. By valuing productivity costs as the steam of lifetime income forgone, a greater weight is given to cancer deaths affecting younger working age males, as opposed to groups with lower labour force participation rates and income including women and the elderly. There have also been assertions that the human capital approach produces results that are either underestimates [32] or overestimates [33] of the true cost. In the absence of a universally agreed ‘best practice’ approach, we determined that the human capital approach was the most appropriate for our purposes given it is the most widely used method in the recent literature [6], therefore enhancing the comparability of our results.

While the truncation of the modelled analysis at 2030 allows for 27 years of future earnings to be accounted for, it does not allow for each individual to be followed until their counterfactual retirement or death. The net impact of this truncation point is likely to be minor, with less than 2% of the total premature death cohort aged 65 years or younger in 2030. In addition, the 3% discount rate applied would necessitate that any costs incurred beyond 2030 be discounted by over 57% of their actual dollar value. Nonetheless, our results may be considered, in this sense, a conservative estimate of the total productivity costs of cancer mortality.

Conversely, the inability of the analysis to explicitly account for the potential correlations between cancer death and common exposures or risk factors among individuals may contribute to an overestimation of the productivity costs. For example, lung cancer deaths occur primarily among smokers, who are also at significantly increased risk of cardiovascular disease [34]. This correlation may be in part addressed by our inclusion of socioeconomic status, in addition to age and sex, as a characteristic upon which mortality records were matched with those of the general population in APPSIM. However, this is unlikely to capture all of the excess mortality risk that could be expected from other causes and as such the analysis may overstate the years of life lost, and in turn the PVLI loss, associated with premature cancer mortality.

Like most developed countries, Australia has an ageing population nearing retirement. The proportion of people aged 65 and over is expected to more than double over the next few decades, resulting in slower workforce and economic growth at the very time that burgeoning demands are placed on the nation’s health and aged care systems. In this context, measures to raise labour force participation and productivity are increasingly being recognised as a key policy focus required to sustain economic growth.

It has been demonstrated that a substantial portion of cancer cases and deaths could be prevented by broadly applying effective prevention measures, such as tobacco control, vaccination, and the use of early detection tests [2]. This paper highlights the significant labour force impacts associated with premature mortality, and in turn indicates the likely economic gains that could accrue with effective interventions. For example, a sustained 2% reduction in lung cancer mortality would lead to long term productivity savings of approximately $15.3M per annum, compounding to result in savings of $841.5M over ten years. Such information can assist decision makers in determining the allocation of resources across competing priorities. This need not be limited to the decisions within a fixed health care budget. For example, an awareness of these costs has the potential to promote the diversion of additional resources to the health care sector away from more traditional measures aimed at increasing productivity, such as labour force or taxation policy.

Premature mortality cost estimates may also be of value in helping set priorities for future research. For example, recent evidence has found that clinical trial activity for individual cancer site interventions in Australia did not align to the relative burden of disease, with four of the five cancers that result in the greatest burden of disease (lung, colorectal, prostate and pancreatic cancers) significantly underrepresented [35]. Similar mismatches in research funding and activity have been reported in the US, UK and Canada [36–38]. Estimates of the relative productivity costs of cancer provides an additional measure of the disease burden that may be used to help inform research funding allocation.

The productivity impacts of premature mortality due to cancer are significant, resulting in 88,000 workings years lost and a cost of $4.2 billion in private income forgone. These results provide an assessment of the relative economic costs of cancer mortality by age and sex, and across multiple cancer sites. The magnitude of these impacts demonstrates the extent of the societal burden of cancer and the potential economic gains that could be achieved through investment in effective interventions. This information may assist decision makers in allocating scare resources amongst competing priorities.

## Supporting Information

S1 Table ICD-10 classifications associated with the cancer types reported. (DOCX)

S2 Table Individual working years lost due to premature cancer mortality by age, sex and cancer type (SAS7BDAT)

S3 Table Individual PVLI lost due to premature cancer mortality by age, sex and cancer type (SAS7BDAT)

## References

[1] Global Burden of Disease Cancer Collaboration. The global burden of cancer 2013. JAMA Oncology. 2015;1(4):505–27. 10.1001/jamaoncol.2015.0735 26181261 PMC4500822

[2] Torre LA, Bray F, Siegel RL, Ferlay J, Lortet-Tieulent J, Jemal A. Global Cancer Statistics, 2012. CA: A Cancer Journal for Clinicians. 2015;65:87–108. 25651787 10.3322/caac.21262

[3] Luengo-Fernandez R, Leal J, Gray A, Sullivan R. Economic burden of cancer across the European Union: a population-based cost analysis. Lancet Oncology. 2013;14:1165–74. 10.1016/S1470-2045(13)70442-X 24131614

[4] Brown ML, Lipscomb J, Snyder C. The Burden of Illness of Cancer: Economic Cost and Quality of Life. Annual Review of Public Health. 2001;22:91–113. 10.1146/annurev.publhealth.22.1.91 11274513

[5] Bradley CJ, Yabroff KR, Dahman B, Feuer EJ, Mariotto A, Brown ML. Productivity Costs of Cancer Mortality in the United States: 2000–2020. Journal of the National Cancer Institute. 2008 100(24):1763–70. 10.1093/jnci/djn384 19066273 PMC2720777

[6] Hanly P, Pearce A, Sharp L. The cost of premature cancer-related mortality: a review and assessment of the evidence. Expert Review of Pharmacoeconomics & Outcomes Research. 2014;14(3):355–77. 24746223 10.1586/14737167.2014.909287

[7] Hanly P, Soerjomataram I, Sharp L. Measuring the societal burden of cancer: The cost of lost productivity due to premature cancer-related mortality in Europe. International Journal of Cancer. 2015;136(4):E136–E45. 10.1002/ijc.29105 25066804

[8] Hanly PA, Sharp L. The cost of lost productivity due to premature cancer-related mortality: an economic measure of the cancer burden. BMC Cancer. 2014;14(224). 10.1186/1471-2407-14-224 PMC3986872 24670067

[9] Kim S-G, Hahm M-I, Choi K-S, Seung N-Y, Shin H-R, Park E-C. The economic burden of cancer in Korea in 2002. European Journal of Cancer Care. 2008;17:136–44. 10.1111/j.1365-2354.2007.00818.x 18302650

[10] Max W, Rice DP, Sung HY, Michel M, Breuer W, Zhang X. The economic burden of gynecologic cancers in California, 1998. Gynecologic Oncology. 2003;88:96–103. 12586586 10.1016/s0090-8258(02)00101-4

[11] Max W, Sung HY, Stark B. The economic burden of breast cancer in California. Breast Cancer Research and Treatment. 2009;116:201–7. 10.1007/s10549-008-0149-4 18683041

[12] Ortiz-Ortiz KJ, Pérez-Irizarry J, Marín-Centeno H, Ortiz AP, Torres-Berrios N, Torres-Cintrón M, et al Productivity Loss in Puerto Rico’s Labor Market due to Cancer Mortality. Puerto Rico Health Sciences Journal. 2010;29(3):241 20799511

[13] Zhang W, Bansback N, Anis A. Measuring and valuing productivity loss due to poor health: A critical review. Social Science and Medicine. 2011;72(2):185–92. 10.1016/j.socscimed.2010.10.026 21146909

[14] Rice DP. Estimating the cost-of-illness Washington, DC: US Department of Health,Education and Welfare, Public Health Service, 1966.

[15] Carter HE, Schofield D, Shrestha R. LifeLossMOD: A microsimulation model of the economic impacts of premature mortality in Australia. International Journal of Mocrosimulation. 2014;7(3):33–52.

[16] International Statistical Classification of Diseases and Related Health Problems, 10th Revision: Version for 2003 [Internet]. 2003. Available from: http://apps.who.int/classifications/apps/icd/icd10online2003/fr-icd.htm.

[17] Australian Bureau of Statistics. Deaths, Australia. Canberra: 2003.

[18] Begg S, Vos T, Barker B, Stevenson C, Stanley L, Lopez AD. The Burden of Disease and Injury in Australia 2003. Canberra: AIHW, 2007.

[19] Kelly S. APPSIM—Selection of the Main Source Data File for the Base Data. Canberra: National Centre for Social and Economic Modelling, University of Canberra, 2007.

[20] Wooden M, Freidin S, Watson N. Enhancing the Evidence Base for Economic and Social Policy in Australia: The Household, Income and Labour Dynamics in Australia (HILDA) Survey. Mercer–Melbourne Institute Quarterly Bulletin of Economic Trends. 2002;3(2):17–20.

[21] Cobb-Clark D. The Longitudinal Survey of Immigrants to Australia. Australian Economic Review. 2001;34(4):467–77.

[22] Pennec S, Bacon B. APPSIM—Modelling Fertility and Mortality in the APPSIM Dynamic Microsimulation Model, Working Paper No. 5. National Centre for Social and Economic Modelling, University of Canberra, 2007.

[23] Fair Work Act. Sect. 62 (2009).

[24] Australian Bureau of Statistics Wage Price Index, Australia, Jun 2015. Canberra: Commonwealth of Australia,; 2015.

[25] Australian Bureau of Statistics Consumer Price Index, Australia, Jun 2015. Canberra: Commonwealth of Australia,; 2015.

[26] Australian Bureau of Statistics. Average Weekly Earnings, Australia, May 2015. Canberra: 2015.

[27] Australian Institute of Health and Welfare. Prostate cancer in Australia. Canberra: AIHW, 2013 Contract No.: Cat. no. CAN 76,.

[28] Australian Bureau of Statistics Labour Force, Australia, Aug 2016. Canberra,: Commonwealth of AUstralia,; 2016.

[29] Keegan M. Modelling the workers of tomorrow: the APPSIM dynamic microsimulation model In: National Centre for Social and Economic Modelling UoC, editor. HILDA Survey Research Conference; Melbourne2007.

[30] Australian Institute of Health and Welfare (AIHW). Australian Burden of Disease Study: Fatal burden of disease 2010. Canberra: 2015.

[31] Carter HE, Schofield D, Shrestha R. The long term productivity impacts of all cause premature mortality in Australia. The Australian and New Zealand Journal of Public Health. 2016;In Press. 10.1111/1753-6405.12604 27868363

[32] Olsen JA, Smith RD. Theory versus practice: a review of ‘willingness-to-pay’ in health and health care. Health Economics. 2001;10(1):39–52. 11180568 10.1002/1099-1050(200101)10:1<39::aid-hec563>3.0.co;2-e

[33] Koopmanschap MA, Rutten FFH. Indirect Costs: The Consequence of Production Loss or Increased Costs of Production Medical Care. 1996;34(12):DS59–DS68 8969315

[34] Campbell SC, Moffatt RJ, Stamford BA. Smoking and smoking cessation—The relationship between cardiovascular disease and lipoprotein metabolism: A review. Atherosclerosis. 2008;201(2):225–35. 10.1016/j.atherosclerosis.2008.04.046 18565528

[35] Dear R, Barratt A, McGeechan K, Askie L, Simes J, Tattersall M. Landscape of cancer clinical trials in Australia: using trial registries to guide future research. Medical Journal of Australia. 2011;194(8):387–91. 21495937 10.5694/j.1326-5377.2011.tb03027.x

[36] Carter A, Nguyen C. A comparison of cancer burden and research spending reveals discrepancies in the distribution of research funding. BMC Public Health. 2012;12. 10.1186/1471-2458-12-526 PMC3411479 22800364

[37] Burnet N, Jefferies S, Benson R, Hunt D, Treasure F. Years of life lost (YLL) from cancer is an important measure of population burden–and should be considered when allocating research funds. British Journal of Cancer. 2005;92:241–5. 10.1038/sj.bjc.6602321 15655548 PMC2361853

[38] Branton P. Does Canadian research investment relate to cancer burden? Lancet Oncology. 2008;9:82–3. 10.1016/S1470-2045(08)70007-X 18237843

