# Supplementary material for: The Productivity Costs of Premature Mortality Due to Cancer in Australia: Evidence from a Microsimulation Model
Source: PLoS One. 2016 Dec 12;11(12):e0167521. doi: 10.1371/journal.pone.0167521 (PMC5152930; doi:10.1371/journal.pone.0167521)
Supplement: S1 Table — (DOCX) [file pone.0167521.s001.docx]

# Appendix 1: ICD-10 classifications associated with the cancer types reported

| **Cancer Type** | **ICD-10 Codes** |
| --- | --- |
| Mouth and oropharynx cancers | C00-C14 |
| Oesophageal cancer | C15 |
| Stomach cancer | C16 |
| Colon and rectum cancers | C18-C21 |
| Liver cancer | C22 |
| Gallbladder cancer | C23 |
| Pancreas cancer | C25 |
| Laryngeal cancer | C32 |
| Bone and connective tissue cancer | C40-C41, C45-C49 |
| Lung cancer | C34 |
| Melanoma | C43 |
| Other skin cancers | C44 |
| Breast cancer | C50 |
| Cervix uteri cancer | C53 |
| Corpus uteri cancer | C54-C55 |
| Ovary cancer | C56 |
| Prostate cancer | C61 |
| Testicular cancer | C62 |
| Kidney cancer | C64 |
| Bladder cancer | C67 |
| Eye cancer | C69 |
| Brain Cancer | C71 |
| Thyroid cancer | C73 |
| Lymphoma | C81-C89, C96 |
| Multiple Myeloma | C90 |
| Leukaemia | C91-C95 |
| Other malignant neoplasms | C17, C23, C24, C26-C31, C33, C37-C40, C51, C52, C57-C60, C63, C65-C66, C68, C70, C72, C74-C80, C97 |
